# HAT1 induces lung cancer cell apoptosis via up regulating Fas

**DOI:** 10.18632/oncotarget.21205

**Published:** 2017-09-23

**Authors:** Na Han, Lei Shi, Qiuyun Guo, Wei Sun, Yang Yu, Li Yang, Xiaoxi Zhang, Mengxian Zhang

**Affiliations:** ^1^ Department of Oncology, Tongji Hospital, Tongji Medical College, Huazhong University of Science and Technology, Wuhan, China

**Keywords:** lung cancer, epithelial cells, HAT1, Fas, apoptosis

## Abstract

The dysfunction of apoptosis is one of the factors contributing to lung cancer (LC) growth. Histone acetyltransferase HAT1 can up regulate cell apoptosis. This study aims to investigate the mechanism by which HAT1 induces LC cell (LCC) apoptosis via up regulating the expression of Fas. In this study, the surgically removed human LC tissues were collected. LCCs were isolated from the LC tissues and analyzed for the expression of HAT1 and Fas by RT-qPCR and Western blotting. We observed that the expression of Fas was negatively correlated with PAR2 in LCCs. Activation of PAR2 suppressed the expression of Fas in normal lung epithelial cells. The expression of HAT1 was lower and positively correlated with Fas expression and negatively correlated with PAR2 expression in LCCs. Activation of PAR2 suppressed Fas expression in lung epithelial cells via inhibiting HAT1. Restoration of HAT1 expression restored Fas expression in LCCs and induced LCC apoptosis. In conclusion, less expression of HAT1 in LCCs was associated with the pathogenesis of LC. Up regulation of HAT1 expression in LCCs can induce LCCs apoptosis, which may be a potential novel therapy for the treatment of LC.

## INTRODUCTION

Lung cancer (LC) is one of the leading diseases of human death [18]. Although the research in this area advanced rapidly in the past several decades, the pathogenesis of LC is still unclear [2]. Despite chemotherapy, radiotherapy and surgery can be used to treat LC, the therapeutic efficacy is unsatisfactory currently; the five-year survival rate of LC is less than 20% [21]. Therefore, it is urgent to invent novel and effective therapies for the treatment of LC. On the other hand, to further investigate the pathogenesis of LC can facilitate to design new and more effective approaches for the management of LC.

Published data indicate that the deregulation of apoptotic mechanism plays a crucial role in the pathogenesis of LC [14]. Apoptosis is also called programmed cell death. The role of apoptosis is to remove senescent cells or damaged cells [4]. Apoptosis in the cells can be regulated by intrinsic or/and extrinsic factors [13]. For example, tumor necrosis factor (TNF), toxic materials, viral infections, and radiations initiate apoptosis [11], while aberrant expression of growth factors may result in deregulation of apoptosis. Although the role of deregulation of apoptosis in LC pathogenesis has been recognized [14], the causative factors are less understood. The management of deregulation of apoptosis remains to be further investigated.

Fas and p53 play an important role in the regulation of apoptosis. P53 is a cancer suppressor protein; it induces cancer cell death via inducing cancer cell apoptosis. Most cancer cells express less p53 or express mutated p53 [12]. Fas is also called CD95, is one of the best studied death receptors. Fas contains a death domain and is involved in the pathogenesis of a number of cancers. To increase the expression of Fas can induce cancer cell death [10]. Yet, the remedies to regulate aberrant Fas expression are still limited.

It is suggested that the histone acetyltransferases (HAT) is associated with the aberrant expression of Fas [15]. The HAT family includes a number of members, which are involved in the gene transcription in the cells. Thus, we hypothesize that the expression of HAT may be aberrant in LCCs, which may compromise the expression of Fas. To test the hypothesis, we collected human LC tissues from the clinic and found that the expression of HAT1, one of the members of HAT family, was markedly lower in LCCs than that in normal lung cells. Further evidence showed that HAT1 was required in the expression of Fas in lung epithelial cells. Restoration of HAT1 restored the expression of Fas in LCCs and induced LCCs apoptosis.

## RESULTS

### Expression of Fas is negatively correlated with PAR2 in LCC

**Figure 1:**
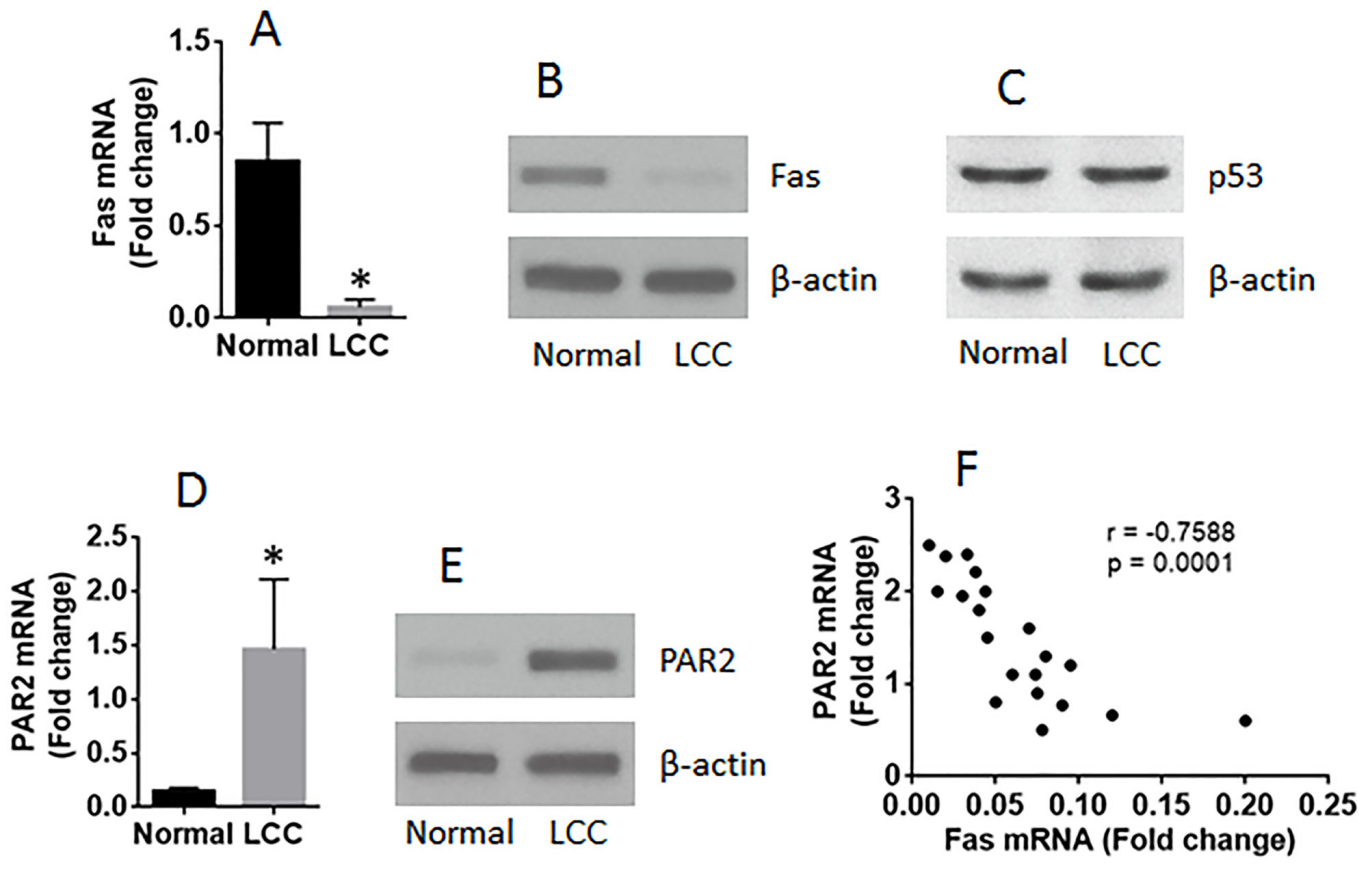
The expression of Fas is negatively correlated with PAR2 expression in LCCs LCCs and normal lung cells were prepared with the surgically removed LC tissue (n=20). The LCCs were analyzed by RT-qPCR and Western blotting. The bars show the mRNA levels of Fas **(A)** and PAR2 **(D)**. The immune blots show the protein levels of Fas **(B)**, p53 **(C)** and PAR2 **(E)**. **(F)** the dot plots show a negative correlation between Fas mRNA and PAR2 mRNA in LCCs. The data of bars are presented as mean ± SD. ^*^p<0.01, compared with the normal group.

LCCs were isolated from surgically removed LC tissues and analyzed by RT-qPCR and Western blotting. The results showed that, as compared the marginal normal lung cells, the expression of Fas was lower (Figure 1A-1B) in LCCs, the levels of p53 was not different between the normal group and the LC group (Figure 1C, and the PAR2 expression was higher in LCCs (Figure 1D-1E). A negative correlation was detected between the expression of Fas and PAR2 in LCCs (Figure 1F).

### Activation of PAR2 suppresses the expression of Fas in normal lung epithelial cells

**Figure 2:**
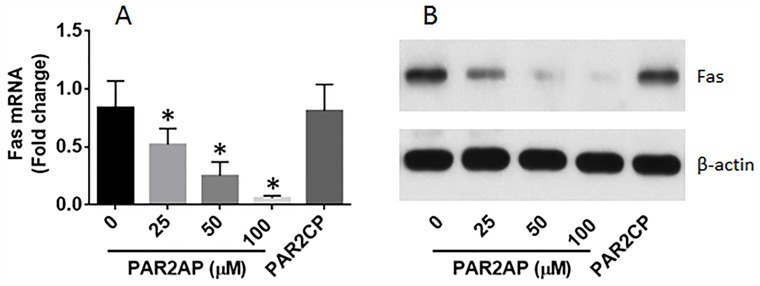
PAR2 suppresses Fas expression in lung cells **(A-B)**, Lung epithelial cells (the BEAS-2B cell line) were cultured in the presence of PAR2 active peptide (PAR2AP) at the indicated concentrations or PAR2 control peptide (PAR2CP; 100 μM) for 48 h. The cells were analyzed by RT-qPCR and Western blotting. The bars indicate the mRNA levels and the immune blots indicate the protein levels of Fas in the normal lung cells. The data of bars are presented as mean ± SD. ^*^p<0.01, compared with the dose “0”. The data represent 3 independent experiments.

The data of Figure 1 imply that PAR2 may be responsible for the suppression of Fas in LCCs. To test this, the BEAS-2B cells, a normal lung epithelial cell line, were exposed to PAR2AP in the culture for 48 h. The cells were then analyzed by RT-qPCR and Western blotting. The results showed that the PAR2 activation did suppress the expression of Fas in lung epithelial cells (Figure 2).

### Expression of HAT1 is positively correlated with Fas expression and negatively correlated with PAR2 expression in LCCs

**Figure 3:**
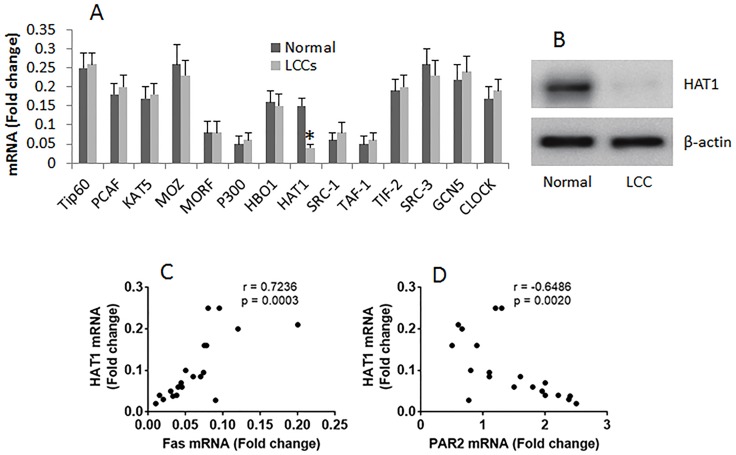
Correlation between HAT1/Fas and HAT1/PAR2 in LCCs The mRNA was extracted from LCCs and normal lung cells, and analyzed by RT-qPCR. **(A)** the bars indicate the mRNA levels of HAT subtypes in LCCs. **(B)** the immune blots indicate the protein levels of HAT1. **(C-D)**, the dot plots show the correlation between HAT1 and Fas (C), HAT1 and PAR2 (D). The data of bars are presented as mean ± SD. ^*^p<0.01, compared with the dose “0”. The data represent 3 independent experiments. The data of Fas and PAR2 used in the correlation test are presented in Figure 1.

The histone acetyltransferases (HAT) are required in gene transcription. Published data indicate that the aberrant expression of HAT is associated with the pathogenesis of cancer [5]. To elucidate if the suppression of Fas in LCCs is associated with aberrant expression of HAT, we screened the expression of subtypes of HAT in LCCs by RT-qPCR. The results showed that the expression of the 14 subtypes of HAT was detectable in all the 20 LC samples, while the expression of HAT1 was uniquely lower than the normal lung cells (Figure 3A-3B). The results imply that the low expression of HAT1 might be associated with lower expression of Fas in LCCs. The inference is supported by performing a correlation test with the data, which showed a positive correlation between HAT1 and Fas, and a negative correlation between HAT1 and PAR2 (Figure 3C-3D).

### Activation of PAR2 suppresses Fas expression in lung epithelial cells via inhibiting HAT1

**Figure 4:**
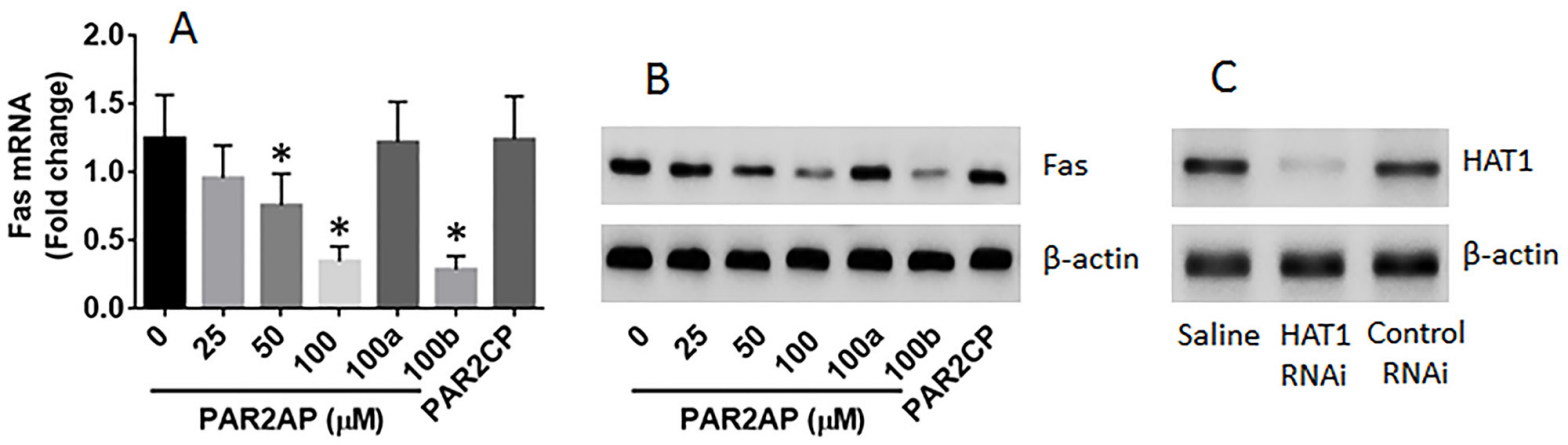
PAR2 suppresses Fas expression in lung epithelial cells Lung epithelial cells (the BEAS-2B cell line) were cultured for 2 days in the presence of PAR2AP at the indicated concentrations. The cells were analyzed by RT-qPCR and Western blotting. **(A-B)**, the mRNA (A) and protein (B) levels of Fas in the lung epithelial cells. 100a (100b): the cells were treated with HAT1 RNAi (control RNAi) and PAR2AP (100 μM). **(C)** the results of HAT1 RNAi. The data of bars are presented as mean ± SD. ^*^p<0.01, compared with the dose “0”. The data represent 3 independent experiments.

**Figure 5:**
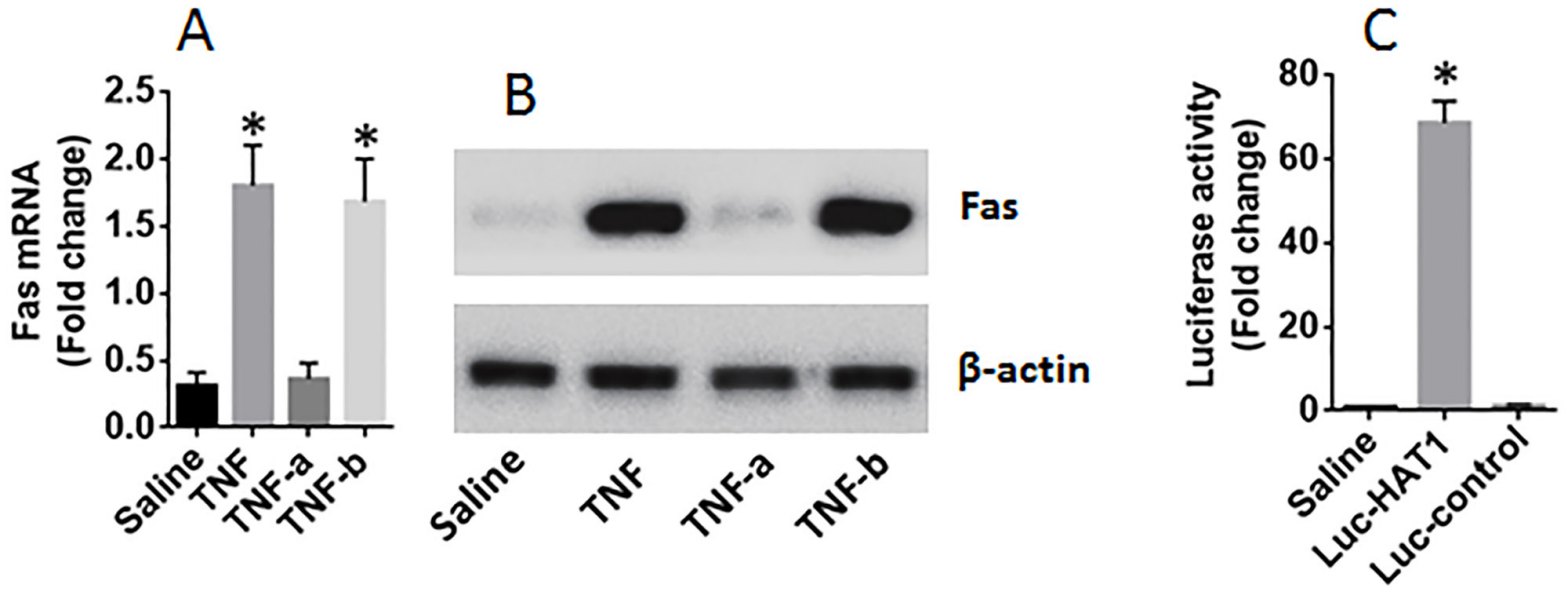
HAT1 is required in the expression of Fas in lung epithelial cells **(A-B)** lung epithelial cells (the BEAS-2B cell line) were exposed to TNF (1 nM) in the culture for 48 h. The cells were analyzed by RT-qPCR and Western blotting. The bars indicate the mRNA levels (A) and the immune blots indicate the protein levels of Fas in the lung epithelial cells. TNF-a (or TNF-b): the cells were treated with HAT1 RNAi (or control RNAi) and TNF at 1 nM. **(C)** HEK293 cells were transfected with Fas promoter plasmids and saline, or a HAT1 luciferase reporter, or a control reporter, respectively. The cells were analyzed by luciferase assay 24 h later. The bars indicate the luciferase activities. The data of bars are presented as mean ± SD. ^*^p<0.01, compared with the saline group. The data represent 3 independent experiments.

Next, lung epithelial cells were exposed to PAR2AP in the culture for 48 h. The cells showed that exposure to PAR2AP in the culture decreased the expression of Fas in the lung epithelial cells in a dose-dependent manner (Figure 4A-4B). To test if HAT1 was involved in the expression of Fas in the lung epithelial cells, the HAT1 were knocked down in the lung epithelial cells by RNAi (Figure 4C). The HAT1-deficient lung epithelial cells were stimulated with PAR2AP in the culture for 48 h. The results showed that knockdown of HAT1 abolished the PAR2AP-decreased Fas expression in lung epithelial cells (Figure 4A-4B). The data suggest that the HAT1 is required in the expression of Fas. To corroborate the results, two more experiments were performed. It is well known that TNF can up regulate the expression of Fas [3]; the lung epithelial cells with or without knocking down the HAT1 gene by RNAi were then exposed to TNF in the culture for 48 h. The results showed that exposure to TNF significantly increased the expression of Fas, which was abolished by knockdown of the HAT1 gene (Figure 5A-5B). In addition, a Fas promoter luciferase reporter and a HAT1 expressing plasmid were constructed and transfected into HEK293 cells. The results of luciferase assay showed the transfection of HAT1 plasmids markedly increased the luciferase activities while the transfection of control plasmids did not (Figure 5C). Together, the results suggest that PAR2 activation does not suppress the expression of Fas directly, but via suppressing HAT1 expression.

### Restoration of HAT1 expression restores Fas expression and induces LCC apoptosis

**Figure 6:**
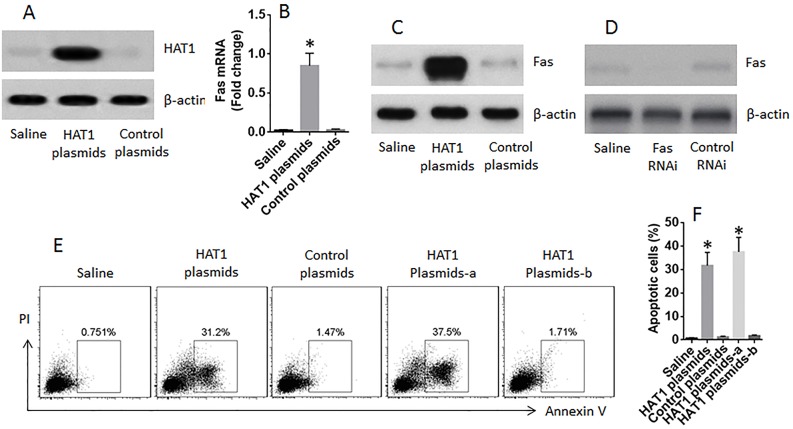
Restoration of HAT1 induces LCCs apoptosis LCCs were prepared from surgically removed LC tissue. **(A)** the immune blots show the expression of HAT1 in LCCs after transfecting with HAT1 plasmids. **(B-C)** the mRNA (B) and protein (C) levels of Fas in LCCs after transfecting with HAT1 plasmids. **(D)** results of Fas RNAi. **(E)** the gated cells are apoptotic LCCs after the treatment denoted above each subpanel and the presence of FasL (1 nM). **(F)** the summarized data of panel E. a: the LCCs were treated with Fas RNAi; b: the LCCs were treated with control RNAi.

The data reported above indicate that HAT1 is involved in the expression of Fas expression in lung epithelial cells. Since the expression of HAT1 and Fas was lower in LCCs, and the Fas is a critical molecule in the initiation of apoptosis, we inferred that restoration of HAT1 might induce LCC apoptosis. To test this, LCCs were prepared; the cells were transfected with HAT1 plasmids, which markedly increased the expression of HAT1 (Figure 6A) and Fas (Figure 6B-6C) in LCCs, as well as induced the LCCs apoptosis; the apoptosis-induction was abolished by knocking down the Fas in LCCs (Figure 6D-6F).

## DISCUSSION

The present study found that the expression of HAT1 and Fas was lower in LCCs than that in normal lung cells. Activation of PAR2 suppressed the expression of HAT1 in lung epithelial cells. HAT1 was required in the expression of Fas. Restoration of HAT1 in LCCs restored the expression of Fas in LCCs and induced LCC apoptosis.

The data showed that the expression of HAT1 was lower in LCCs. HAT1 is one of the members of HAT. As the important factors in gene transcription, the role of HATs in the pathogenesis of cancer has been extensively studied. It is suggested that HATs drive cellular plasticity in the cell reprogramming and tumorigenesis [9]. The activities of HAT link the different factor-induced acetylation signaling in genome maintenance and cancer [7]. Previous studies found that HATs are associated with the pathogenesis of cancers; but most of them demonstrated that the expression of some members of HAT was higher in cancer cells. Such as HAT hMOF involves tumorigenesis in lung cancer by promoting S phase entry [22] and the expression of GCN5 is elevated in colon cancer cells [20]. Our data show a decrease in HAT in cancer, the levels of HAT1 were markedly lower in LCCs; this is in line with published data; Simo-Riudalbas et al reported that the expression of HAT6b was lower in non-small cell LC [16]. Thus, the expression of HAT members in cancer can be various depending on individual cancers.

The data show a positive correlation between the expression of HAT1 and Fas in LCCs. Fas is an important molecule in the initiation of apoptosis. Cumulative reports indicate the deregulation of Fas expression in Cancer. Such as He et al found that the expression of Fas was lower in pancreatic cancer. The levels of Fas expression were significantly associated with better outcome of pancreatic cancer [8]. Wang et al observed that the expression of CD95 was lower in liver cancer and negatively correlated with histological differentiation, liver cirrhosis, lymph node metastasis and distant metastasis [19]. The present data add further information in the study of Fas that the lower expression of HAT1 is positively correlated with the expression of Fas in LCCs. HAT1 is one of the HAT members, which involve in the gene transcription. The data indicate that the lower expression of HAT1 could be one of the causative factors of the lower expression of Fas in LCCs. The inference is corroborated by further data that the HAT1 is required in the induction of Fas in lung epithelial cells.

Although the deregulation of Fas expression has recognized in cancers, the causative factors of Fas suppression are not fully elucidated. The data show that the activation of PAR2 suppresses expression of HAT1 in lung epithelial cells, indicating that factors activating PAR2 may suppress Fas expression and contribute to the pathogenesis of cancer. Such as mast cell activation can activate PAR2 by releasing tryptase; the latter cleaves the exterior part of PAR2 molecule and activates PAR2. Mast cells distribute extensively in the body. Multiple factors, such as allergic reactions, can activate mast cells to induce mast cells to release chemical mediators, like tryptase. Whether mast cells play a critical role in the pathogenesis via suppressing Fas expression needs to be further investigated. This inference is supported by previous studies [1]. By immunohistochemistry, Globa et al found that the intratumoral mast cell phenotype of malignant tumor was tryptase+/chymase+/CD117+, while in the peritumoral areas three different MCs phenotypes were identified: tryptase+/chymase+/CD117-, tryptase+/CD117+/chymase- and chymase+/CD117+/tryptase- [6]. Stoyanove et al found that mast cell-derived histamine promoted non-small cell LCC proliferation [17].

P53 is also a tumor suppressor. In the present study, we also observed p53 in LCCs, but the levels of p53 in LCCS were not significantly different from the normal controls. We also did not identify the correlation between p53 and HAT1 in LCCs. Thus, HAT1 may not regulate the expression of p53 in LCCs.

In summary, the present study found that the expression of Fas and HAT1 was lower in LCCs. Activation of PAR2 suppressed the expression of HAT1 in lung epithelial cells. HAT1 was required in the expression of Fas. Restoration of HAT1 increased the expression of Fas and induced LCCs apoptosis.

## MATERIALS AND METHODS

### Reagents

The RNAi kits of HAT1 and Fas, antibodies of Fas and HAT1 were purchased from Santa Cruz Biotech (Santa Cruz, CA). The recombinant TNF, FasL, PAR2-activating peptides SLIGRL and control peptide LRGILS were purchased from R&D Systems (Minneapolis, MN). The Annexin V kit and propidium iodide were purchased from Sigma Aldrich (St. Louis., MO). The reagents for performing RT-qPCR, Western blotting and gene transfection were purchased from Invitrogen (Carlsbad, CA). Immune cell isolation reagent kits were purchased from Miltenyi Biotech (San Diego, CA).

### Collection of LC tissue

Surgically removed LC tissues were collected from the operation room of the Wuhan Tongji Hospital. The LC tissues were from 20 LC patients (male 10 and female 10; age: 35 to 66 years old). The diagnosis and management of LC were carried out by our surgeons and pathologists. Patients had one of the following conditions were excluded from the study: LC with metastasis; treated with anti-cancer therapy; smoking history, suffered with other immune disorders or severe organ diseases. The study plan was approved by the Human Ethics Committee of Tongji Hospital. A written informed consent was obtained from each human subject.

### Preparation of LCCs

The LC tissues were cut into small pieces (2×2×2 mm) and incubated for 2 h in the presence of a collagenase IV (1 mg/ml) at 37 °C with mild agitation. The cells were filtered through cell strainers (100 μm first, then 40 μm). Immune cells (CD3^+^, CD11c/b^+^, CD19^+^, CD14^+^, CD68^+^, CD117^+^) were selected out by magnetic cell sorting (MACS) with commercial reagent kits following the manufacturer's instructions. The remained cells were used as LCCs. Cells isolated from the marginal normal tissue (approved by pathology) were used as normal lung cells.

### Cell culture

The isolated LCCs, normal lung cells and BEAS-2B cells (a lung epithelial cell line; ATCC) were cultured in DMEM supplemented with 10% fetal bovine serum, 2 mM L-glutamine, antibiotics (100 U/ml penicillin and 0.1 mg/ml streptomycin). The medium was changed in 1-2 days. The cell viability was greater than 98% as assessed by trypan blue exclusion assay.

### Real time quantitative RT-PCR (RT-qPCR)

The total RNA was extracted from cells with the TRIzol reagents. The cDNA was synthesized with a reverse transcription reagent kit following the manufacturer's instructions. The samples were amplified in a real time PCR device with the SYBR Green Master Mix in the presence of primers as indicated in Table 1. The results are presented in fold change against the internal housekeeping gene β-actin.

**Table 1:** Primers used in the present study

Molecule	Forward	Reverse
PCAF	cctcttctggacttgaggca	tggtctctggtccaagcatt
HAT1	ctacaatgtggcctgcatcc	ttgatggtgatctgtggcct
MOZ	tgcccaatcctttctaagcc	acactacttactgagcgccg
MORF	cttaatggactgtggcgtcg	ctgctctcctgggtcaaaga
P300	ttgtgaagagccccatggat	gctttgcatcactgggtcaa
HBO1	ccacgaactcctcctccatt	agtcagggattgtggtgagg
HAT1	gtgtacccagacaaaacccg	ccgggaaaaacagggcaaat
SRC-1	ggtaaaaggtgcaggtgtgg	acaaagtacggggaaggagg
TAF-1	aagttcctgggcctaactcc	ctcttccggatgctgctttc
TIF-2	gttttgccagccacttacca	aacacactggcgttttctcc
SRC-3	acatgggagtcctggtcttg	ctggaaaagctgtggttccc
GCN5	tccccattgacctgaagacc	cctccctccttgagcttgaa
CLOCK	gtgttacatcgcgccatcat	cattaggagggctgagaggg
Fas	cttctggggagtgagggaag	cttccccaactccgtactga
PAR2	gcgatcttctgccatggatg	gtcgatgcagctgttaaggg

### Western blotting

The total protein was extracted from cells, separated by SDS-PAGE (Sodium dodecyl sulfate polyacrylamide gel electrophoresis) and transferred onto a PVDF membrane. The membrane was blocked by incubating with 5% skim milk for 30 min, stained with the primary antibodies of interest overnight at 4 °C, washed with TBST (Tris-buffered saline Tween 20), incubated with the secondary antibodies (labeled with peroxidase) for 1 h at room temperature and washed with TBST again. The immune blots were developed by the enhanced chemiluminescence and photographed with an imaging device.

### RNA interference (RNAi)

The HAT1 gene or the Fas gene was knocked down from lung epithelial cells or LCCs by RNAi with commercial reagent kits following the manufacturer's instructions. The effects of RNAi were assessed by Western blotting 48 h after RNAi.

### Luciferase assay

DNA fragments of the Fas promoter (tagged with luciferase) and the HAT1 gene-containing plasmids were synthesized by the Sangon Biotech (Shanghai, China). HEK293 cells (1 × 10^5^ per well) were transfected with 1 μg Fas promoter luciferase reporter and 1 μg HAT1 plasmids together with Lipofectamine 2000 (Invitrogen), following the manufacturer's instructions. Luciferase activities were assessed 48 h after transfection with a luciferase reporter assay kit (Promega).

### Restoration of HAT1 expression in LCCs

LCCs were prepared from the surgically removed LC tissues as described above. The LCCs were transfected with the HAT1 plasmids following the manufacturer's instructions. The effects of transfection were assessed by Western blotting 48 h after the transfection.

### Assessment of apoptosis in LCCs

LCCs were prepared as described above and stained with propidium iodide (PI; 5 μg/ml) and an annexin V kit following the manufacturer's instructions. The cells were then analyzed by flow cytometry. The annexin V^+^ PI^+^ or annexin V^+^ PI^-^ cells were regarded as apoptotic cells.

### Statistics

The data are presented as mean ± SD. The differences between two groups were determined by Student t test or ANOVA if more than two groups. P<0.05 was set as a significant criterion.

## References

[1] de Souza Junior DA, Santana AC, da Silva EZ, Oliver C, Jamur MC (2015). The Role of Mast Cell Specific Chymases and Tryptases in Tumor Angiogenesis. Biomed Res Int.

[2] Dela Cruz CS, Tanoue LT, Matthay RA (2011). Lung cancer: epidemiology, etiology, and prevention. Clin Chest Med.

[3] Dong J, Cui X, Jiang Z, Sun J (2013). MicroRNA-23a modulates tumor necrosis factor-alpha-induced osteoblasts apoptosis by directly targeting Fas. J Cell Biochem.

[4] Elmore S (2007). Apoptosis: a review of programmed cell death. Toxicol Pathol.

[5] Farria A, Li W, Dent SY (2015). KATs in cancer: functions and therapies. Oncogene.

[6] Globa T, Saptefrti L, Ceausu RA, Gaje P, Cimpean AM, Raica M (2014). Mast cell phenotype in benign and malignant tumors of the prostate. Pol J Pathol.

[7] Gong F, Chiu LY, Miller KM (2016). Acetylation Reader Proteins: Linking Acetylation Signaling to Genome Maintenance and Cancer. PLoS Genet.

[8] He C, Jiang H, Geng S, Sheng H, Shen X, Zhang X, Zhu S, Chen X, Yang C, Gao H (2014). Expression and prognostic value of c-Myc and Fas (CD95/APO1) in patients with pancreatic cancer. Int J Clin Exp Pathol.

[9] Hirsch CL, Wrana JL, Dent SYR (2017). KATapulting toward Pluripotency and Cancer. J Mol Biol.

[10] Ivanov VN, Ronai Z, Hei TK (2006). Opposite roles of FAP-1 and dynamin in the regulation of Fas (CD95) translocation to the cell surface and susceptibility to Fas ligand-mediated apoptosis. J Biol Chem.

[11] Kiraz Y, Adan A, Kartal Yandim M, Baran Y (2016). Major apoptotic mechanisms and genes involved in apoptosis. Tumour Biol.

[12] Meek DW (2015). Regulation of the p53 response and its relationship to cancer. Biochem J.

[13] Ola MS, Nawaz M, Ahsan H (2011). Role of Bcl-2 family proteins and caspases in the regulation of apoptosis. Mol Cell Biochem.

[14] Pore MM, Hiltermann TJ, Kruyt FA (2013). Targeting apoptosis pathways in lung cancer. Cancer Lett.

[15] Ren YB, Luo T, Li J, Fu J, Wang Q, Cao GW, Chen Y, Wang HY (2015). p28(GANK) associates with p300 to attenuate the acetylation of RelA. Mol Carcinog.

[16] Simo-Riudalbas L, Perez-Salvia M, Setien F, Villanueva A, Moutinho C, Martinez-Cardus A, Moran S, Berdasco M, Gomez A, Vidal E, Soler M, Heyn H, Vaquero A (2015). KAT6B Is a Tumor Suppressor Histone H3 Lysine 23 Acetyltransferase Undergoing Genomic Loss in Small Cell Lung Cancer. Cancer Res.

[17] Stoyanov E, Uddin M, Mankuta D, Dubinett SM, Levi-Schaffer F (2012). Mast cells and histamine enhance the proliferation of non-small cell lung cancer cells. Lung Cancer.

[18] Tanoue LT, Tanner NT, Gould MK, Silvestri GA (2015). Lung cancer screening. Am J Respir Crit Care Med.

[19] Wang X, Wei K, Zhang Q, Zeng S, Lin J, Qiao L, Liu L (2015). Expression of cluster of differentiation-95 and relevant signaling molecules in liver cancer. Mol Med Rep.

[20] Yin YW, Jin HJ, Zhao W, Gao B, Fang J, Wei J, Zhang DD, Zhang J, Fang D (2015). The Histone Acetyltransferase GCN5 Expression Is Elevated and Regulated by c-Myc and E2F1 Transcription Factors in Human Colon Cancer. Gene Expr.

[21] Yu G, Herazo-Maya JD, Nukui T, Romkes M, Parwani A, Juan-Guardela BM, Robertson J, Gauldie J, Siegfried JM, Kaminski N, Kass DJ (2014). Matrix Metalloproteinase-19 Promotes Metastatic Behavior In Vitro and Is Associated with Increased Mortality in Non-Small Cell Lung Cancer. Am J Respir Crit Care Med.

[22] Zhao L, Wang DL, Liu Y, Chen S, Sun FL (2013). Histone acetyltransferase hMOF promotes S phase entry and tumorigenesis in lung cancer. Cell Signal.

